# Effects of endoluminal vacuum sponge therapy on the perfusion of gastric conduit in a porcine model for esophagectomy

**DOI:** 10.1007/s00464-023-10647-0

**Published:** 2024-01-05

**Authors:** Eleni Amelia Felinska, Alexander Studier-Fischer, Berkin Özdemir, Estelle Willuth, Philipp Anthony Wise, Beat Müller-Stich, Felix Nickel

**Affiliations:** 1 Department of General, Visceral and Transplant Surgery, University of Heidelberg, Heidelberg, Germany; 2 Department of Surgery, Clarunis University Center for Gastrointestinal and Liver Disease, University Hospital and St. Clara Hospital Basel, Basel, Switzerland; 3 Department of General, Visceral, and Thoracic Surgery, University Medical Center Hamburg Eppendorf, Martinistrasse 52, 20246 Hamburg, Germany

**Keywords:** Endoluminal vacuum therapy, Esophagectomy, Anastomotic leak, Hyperspectral imaging, Perfusion, Gastric conduit

## Abstract

**Background:**

After esophagectomy, the postoperative rate of anastomotic leakage is up to 30% and is the main driver of postoperative morbidity. Contemporary management includes endoluminal vacuum sponge therapy (EndoVAC) with good success rates. Vacuum therapy improves tissue perfusion in superficial wounds, but this has not been shown for gastric conduits. This study aimed to assess gastric conduit perfusion with EndoVAC in a porcine model for esophagectomy.

**Material and methods:**

A porcine model (n = 18) was used with gastric conduit formation and induction of ischemia at the cranial end of the gastric conduit with measurement of tissue perfusion over time. In three experimental groups EndoVAC therapy was then used in the gastric conduit (− 40, − 125, and − 200 mmHg). Changes in tissue perfusion and tissue edema were assessed using hyperspectral imaging. The study was approved by local authorities (Project License G-333/19, G-67/22).

**Results:**

Induction of ischemia led to significant reduction of tissue oxygenation from 65.1 ± 2.5% to 44.7 ± 5.5% (p < 0.01). After EndoVAC therapy with − 125 mmHg a significant increase in tissue oxygenation to 61.9 ± 5.5% was seen after 60 min and stayed stable after 120 min (62.9 ± 9.4%, *p* < 0.01 vs tissue ischemia). A similar improvement was seen with EndoVAC therapy at − 200 mmHg. A nonsignificant increase in oxygenation levels was also seen after therapy with − 40 mmHg, from 46.3 ± 3.4% to 52.5 ± 4.3% and 53.9 ± 8.1% after 60 and 120 min respectively (*p* > 0.05). An increase in tissue edema was observed after 60 and 120 min of EndoVAC therapy with − 200 mmHg but not with − 40 and − 125 mmHg.

**Conclusions:**

EndoVAC therapy with a pressure of − 125 mmHg significantly increased tissue perfusion of ischemic gastric conduit*.* With better understanding of underlying physiology the optimal use of EndoVAC therapy can be determined including a possible preemptive use for gastric conduits with impaired arterial perfusion or venous congestion.

**Graphical abstract:**

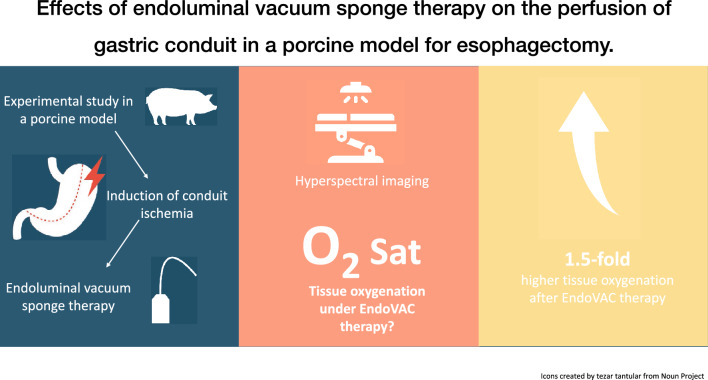

**Supplementary Information:**

The online version contains supplementary material available at 10.1007/s00464-023-10647-0.

Esophageal cancer is the ninth most common cancer in the world, with the number of cases rising in industrialized countries [1]. Potentially curative treatment involves esophagectomy, which is considered a high-risk procedure. The 5-year survival rate is 20–40% and in 40–80% of the cases postoperative complications occur, of which the anastomotic leakage (AL) is the main driver of morbidity and mortality [2]. Despite optimization of the anastomotic technique, the rate of AL is still high, varying between 5 and 30% [2]. In addition, this dreaded complication increases the length of hospital stay, delays the transition to a normal diet, and increases the risk of strictures or revisional surgery [3–6]. AL is associated with a reduced quality of life and has a negative impact on long-term survival [7–9]. Complication management of AL after esophagectomy can consist of conservative therapy, endoscopic intervention, radiologic intervention, and surgical intervention [10]. The most commonly used procedures are endoscopic stent placement and endoluminal vacuum therapy (EndoVAC) and recent clinical evidence shows good results for EndoVAC in management of AL [11–24]. As a result, some clinics even describe establishing a preemptive EndoVAC therapy after esophagectomy for high-risk patients, or even independently from the patient’s risk profile [25]. However, very little is known about the physiological basis of EndoVAC and its effects and the results of available studies show some discrepancies. In 1997, Morykwas et al. first described in a series of animal experiments a novel subatmospheric pressure technique for wound treatment, the vacuum assisted closure (VAC), which significantly increased blood flow levels, rates of granulation tissue formation, and clearance of bacteria from infected superficial wounds [26]. In addition to increased blood flow, the reduction of tissue edema after VAC therapy has been described [26–28]. The available studies all investigated superficial wound treatment and there is no evidence that these mechanisms of action are applicable also to EndoVAC therapy of internal organs.

In general, AL is of a multifactorial nature. More than twenty risk factors varying from the surgical techniques to individual patients characteristics such as diabetes or overweight have been recently defined [29–33]. Interestingly, risk factors directly associated with perfusion of the gastric conduit, such as calcification of celiac axis or intraoperative hypotension, increased the AL risk by three to four-fold [29]. Therefore, the aim of the current study was to investigate systematically the effects of EndoVAC therapy on gastric conduit perfusion with different suction settings on gastric conduit tissue, and after inducing ischemia in the anastomotic area by interrupting the arterial blood supply or inducing venous congestion.

## Materials and methods

### Study design

**Fig. 1:**
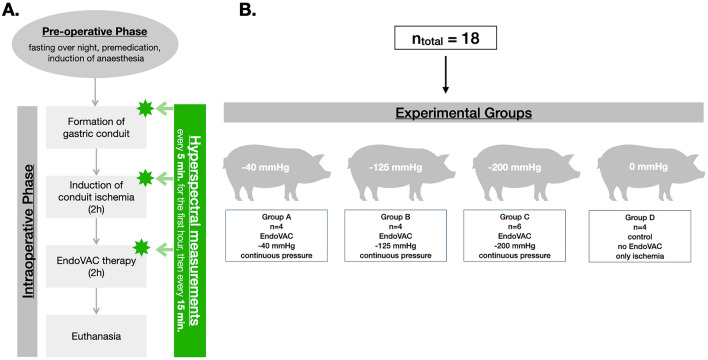
Experimental workflow. **A** The preoperative phase consisted of the preparation of the gastrointestinal tract by 24-h fasting period and the induction of anesthesia. The intraoperative procedure consisted of formation of gastric conduit, induction of conduit ischemia, and implementation of EndoVAC therapy. Intraoperatively, hyperspectral measurements were made as depicted in the figure. **B** A total of 18 piglets were divided into four experimental groups. Different suction settings of the EndoVAC therapy were tested in each group including − 40 mmHg, − 125 mmHg, and − 200 mmHg continuous pressure. Control group did not receive any EndoVAC therapy

The EndoVAC study was a surgical trial with 18 piglets undergoing gastric conduit formation and induction of conduit ischemia (Fig. 1.). Piglets were divided into three experimental groups that received an EndoVAC therapy regimen differing in the level of negative pressure. Examined negative pressure levels were 40 mmHg, 125 mmHg, and 200 mmHg for group A, B, and C, respectively. The control group (group D, *n* = 4) underwent conduit ischemia for the entire period of the experiment without consecutive EndoVAC therapy. Piglets (Large White, mean weight: 35.6 ± 4.8 kg) were supplied by the local farmer. All experiments were conducted by the same surgeon.

### Experimental procedure

After a preoperative fasting period of 24 h without withholding water, the premedication was administered approximately 60 min before surgery, with intramuscular azaperone (6 mg/kg) followed by ketamine (20 mg/kg) with midazolam (0,75 mg/kg). Intravenous propofol (3 mg/kg) was used for anesthesia induction prior to endotracheal intubation. Inhalational anesthesia was maintained with 2% sevoflurane and intravenous ketamine with midazolam for analgesia.

After performing a median laparotomy, the stomach was first mobilized through dividing the gastric vessels of the lesser curvature and dissecting the paraesophageal region by using a monopolar vessel-sealing device (LigaSure Maryland™, Medtronic, USA). The right gastroepiploic artery was maintained at all times. Next, the esophagus was disconnected 1 cm above the gastroesophageal junction and a 56-French bougie introduced through the incision. Starting at the lesser curvature, the gastric conduit (GC) was created along the 56-Fr bougie using a stapling device (ENDO GIA™ equipped with 60 mm Blue Reloads, Medtronic, USA). Subsequently, the previously prepared sponge connected to a vacuum device (Invia Liberty, Medela Medizintechnik GmbH & Co. Handels KG, Eching, Germany) was placed in the gastric lumen and the tissue ischemia was induced toward the lesser curvature/staple line by disrupting the microcirculation by strong magnet compression of the tissue at the greater curvature parallel to the gastroepiploic vessels distally as previously described [34]. The incision was closed with 3–0 sutures to avoid loss of pressure during the subsequent EndoVAC therapy. After 2 h, the continuous negative pressure was switched on and left for another 2 h (Fig. 1). The animals were then euthanized by intravenous administration of 50 ml potassium chloride (7.45%)(Supplementary file 1.).

### Intraoperative hyperspectral measurements

The measurements were done with the hyperspectral imaging system TIVITA® Tissue (Diaspective Vision GmbH, Germany) at different time points depicted in Fig. 1. To acquire hyperspectral images the hyperspectral imager was placed 35–40 cm above the porcine stomach, the ambient lights switched off and hyperspectral images were acquired with the camera-integrated software. The following parameters were recorded: (1) tissue oxygenation (StO2 [%]), tissue hemoglobin index (THI), Near-infrared perfusion index (NIR), and tissue water index (TWI).

### Statistical methods

Raw data were obtained and analyzed using the annotation software [35, 36]. Then, data were entered into a spreadsheet and the statistical evaluation was done with GraphPad Prism version 9.2.0. for Mac (GraphPad Software, San Diego, California, USA). A *p*-value ≤ 0.05 was considered statistically significant. In case of parametric data, paired and unpaired t-test was used. For comparisons of multiple groups over the time, one-way ANOVA was used in case of parametric normal distribution.

## Results

### Induction and maintenance of tissue ischemia

**Fig. 2:**
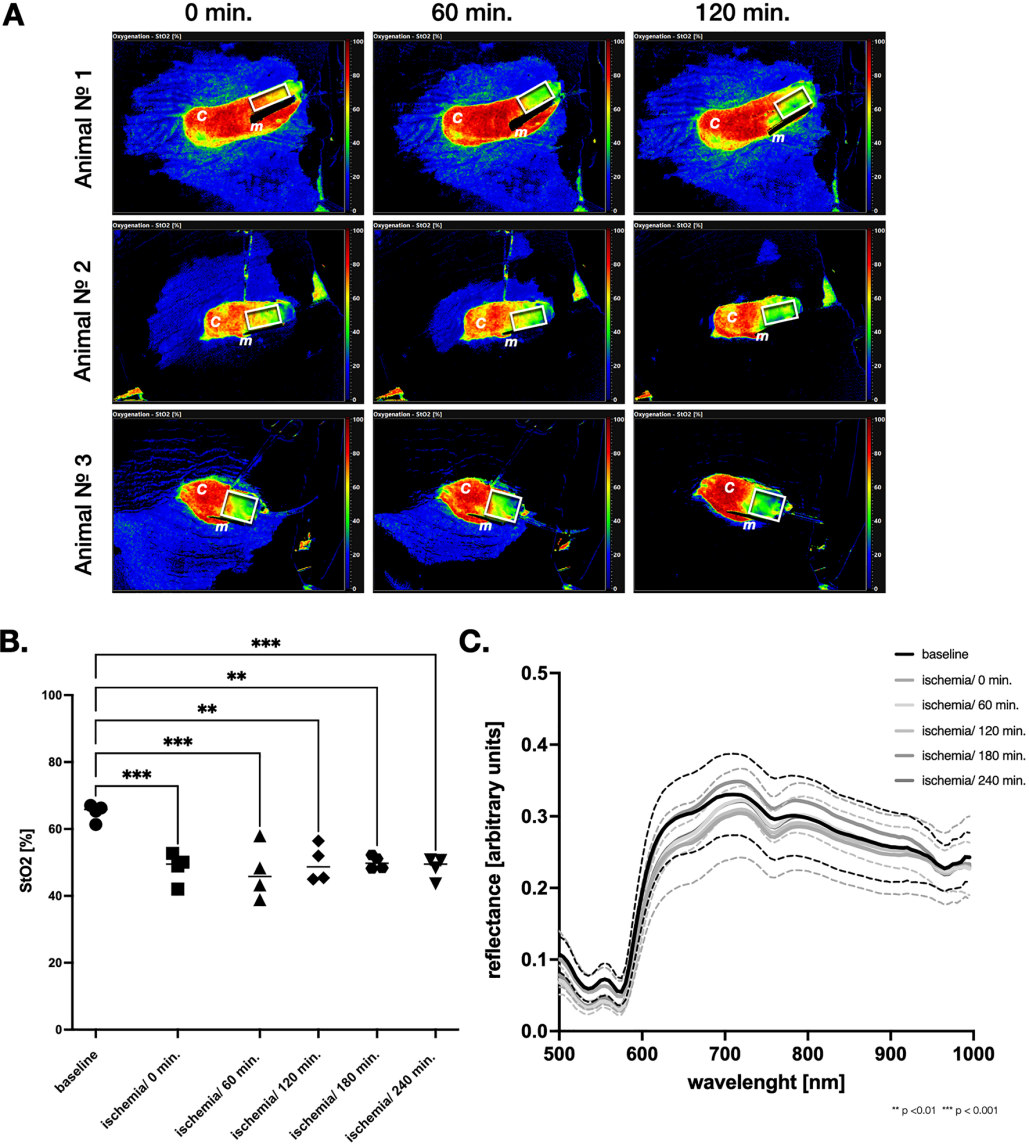
Induction and maintenance of tissue ischemia. **A** Tissue ischemia was induced by disrupting the microcirculation by strong magnet compression of the tissue for total of 120 min in the experimental group. **B** In the control group tissue ischemia was maintained for the entire duration of the experiment. There was no spontaneous increase in tissue oxygenation (StO2%) without EndoVAC therapy after 240 min. **C** Relative reflectance confirmed the measured results. *c* conduit, *m* magnet, white box indicates the region of interest

A stable and pronounced hypoperfusion of the cranial region of gastric conduit was achieved after magnet compression after 120 min. The oxygenation index dropped from 65.1 ± 2.5% to 47.1 ± 8.2% and 49.7 ± 5.5% after 60 min. and 120 min., respectively (Fig. 2.). The hypoperfusion of the gastric conduit could be managed for the entire time of the experiment and remained stable at 48.4 ± 3.3% after 240 min in the control group (Fig. 2 and Table 1).

**Table 1:** Tissue Oxygenation Index (StO_2_%)

	Group A	Group B	Group C		Group D
	(− 40 mmHg)	(− 125 mmHg)	(− 200 mmHg)		(no EndoVAC)
*n*	4	4	6	*n*	4
Baseline	65.6 ± 4.3	66.6 ± 2.5	68.7 ± 3.7	Baseline	65.1 ± 2.5
Tissue ischemia after				Tissue ischemia after	
60 min	45.4 ± 4.5	43.3 ± 6.1	42.8 ± 3.9	60 min	47.1 ± 8.2
120 min	46.3 ± 3.4	44.9 ± 7.6	41.7 ± 5.2	120 min	49.7 ± 5.5
EndoVAC after 60 min	52.5 ± 4.3	61.9 ± 5.5	65.3 ± 3.1	180 min	49.9 ± 2.1
EndoVAC after 120 min	53.9 ± 8.1	62.9 ± 9.4	67.6 ± 3.4	240 min	48.4 ± 3.3

Mean values and standard deviation for each experimental group are depicted in the table below

### The tissue oxygenation depended on the level of applied negative pressure

**Fig. 3:**
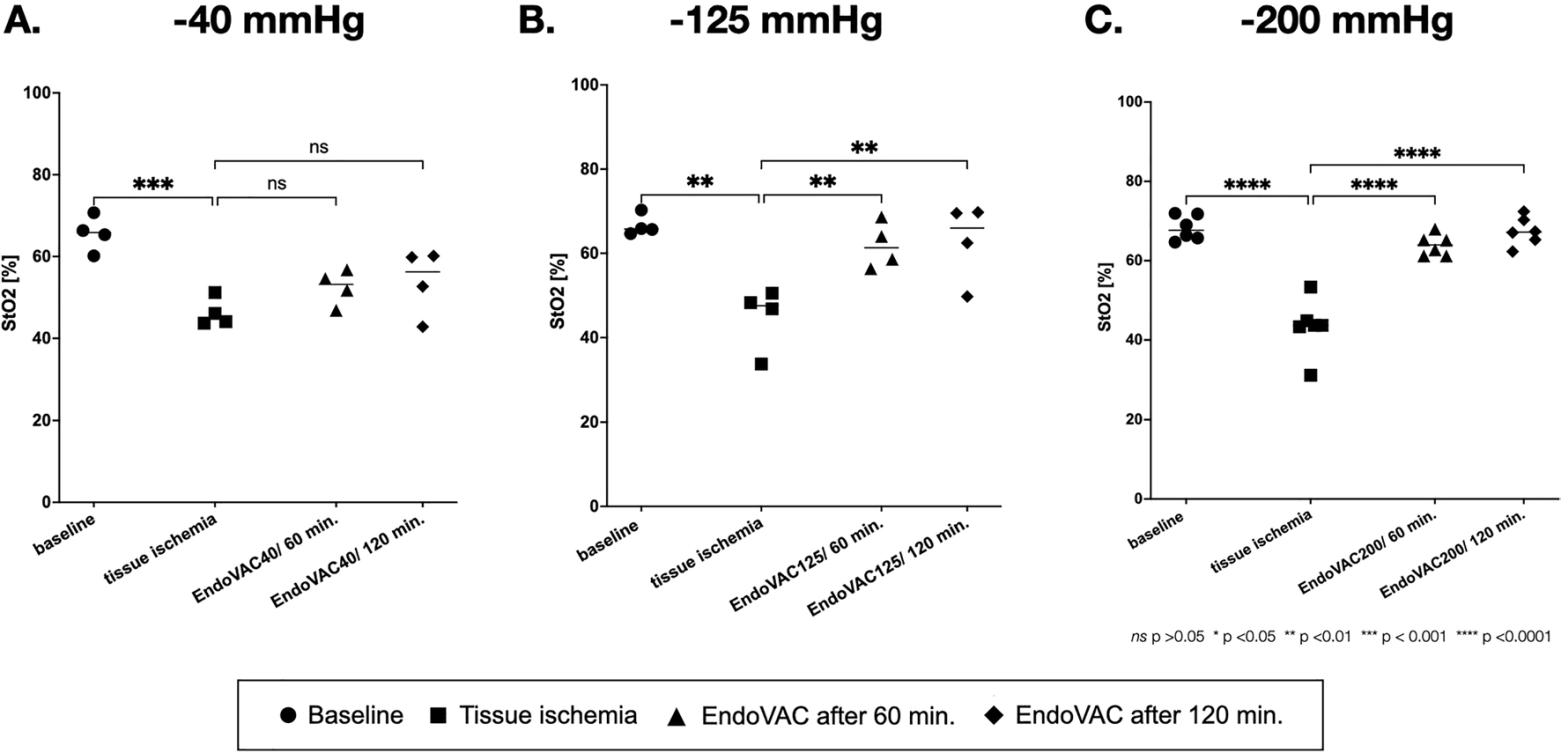
Tissue oxygenation after EndoVAC therapy. **A** Tissue oxygenation under EndoVAC therapy with − 40 mmHg pressure did not significantly improve after 60 and 120 min. **B** and **C** Under EndoVAC therapy with − 125 mmHg and − 200 mmHg pressure, tissue oxygenation improved significantly and reached after 120 min values around 62% and 68%, respectively. The points represent individual animals

Tissue oxygenation under EndoVAC therapy with − 40 mmHg pressure did not significantly improve after 60 and 120 min. The oxygenation index was not significantly changed from 46.3 ± 3.4% to 52.5 ± 4.3% (*p* = 0.26) and 53.9 ± 8.1% (*p* = 0.15), respectively. The oxygenation index did not return to the baseline level of 65.6 ± 4.3% (Fig. 3 and Table 1).

After EndoVAC therapy with − 125 mmHg a significant increase in the oxygenation index from 44.9 ± 7.6% to 61.9 ± 5.5% (*p* < 0.01) was seen after 60 min. After 120 min, the oxygenation level stayed stable at 62.9 ± 9.4% and was still significantly increased compared to tissue ischemia (*p* < 0.01) (Fig. 3 and Table 1).

A similar improvement was seen under the EndoVAC therapy with − 200 mmHg. The oxygenation index significantly increased from 41.7 ± 5.2% to 65.3 ± 3.1% (*p* < 0.0001) and 67.6 ± 3.4% (*p* < 0.0001) after 60 and 120 min, respectively, reaching nearly the baseline levels of 68.7 ± 3.7% (Fig. 3 and Table 1).

**Fig. 4:**
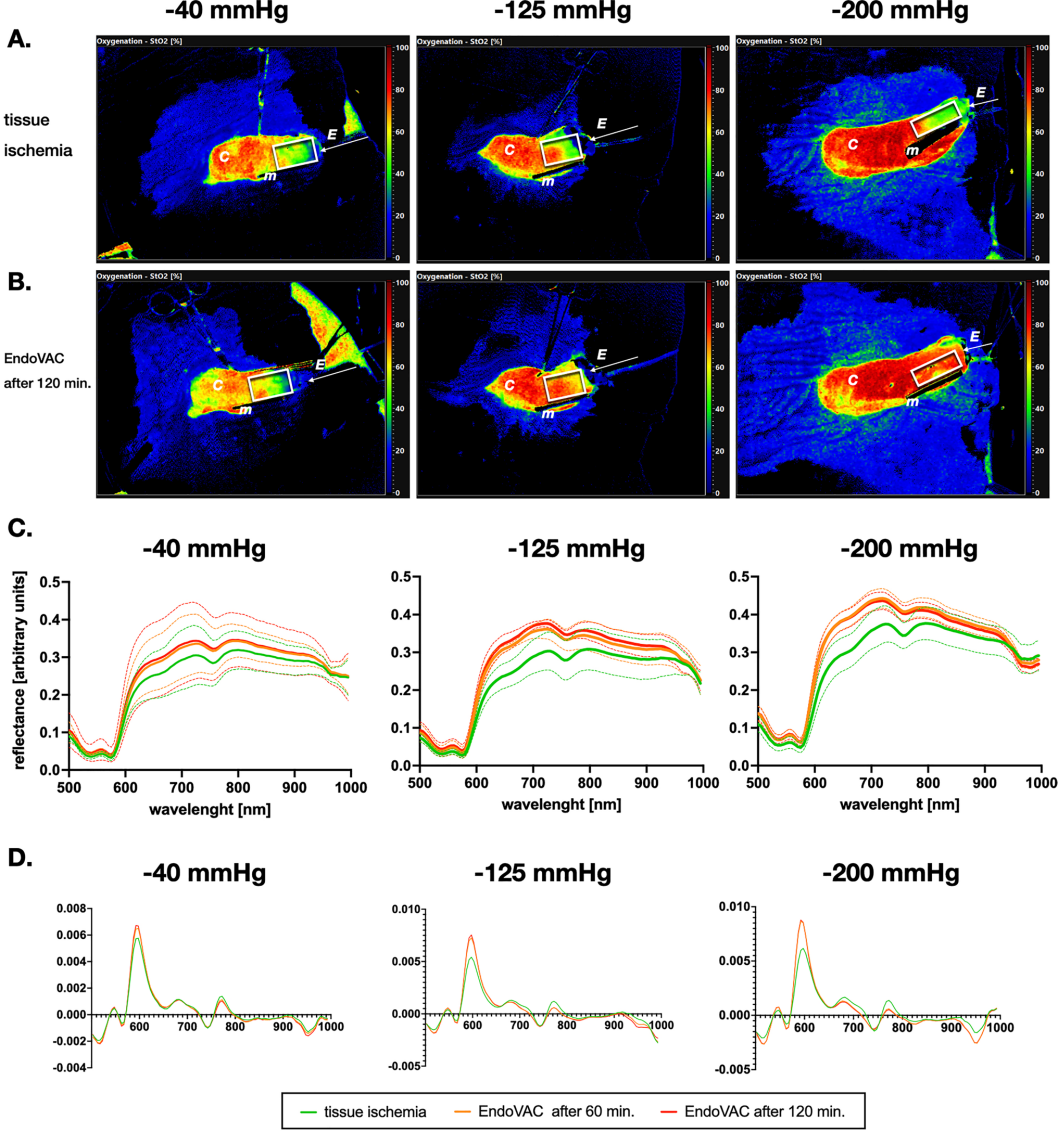
Hyperspectral representation of tissue oxygenation. The hyperspectral images show the gastric conduit **A** after induction of tissue ischemia and **B** after EndoVAC therapy for 120 min with pressure of − 40, − 125, and − 200 mmHg. The green and red areas correspond to a poor and good oxygenation index respectively. **C** Relative reflectance intensities and **D** their first derivatives show distinct changes depicting the oxygenation status of the hemoglobin (wavelengths between 550 and 600 nm and above 700 nm) and the water content (wavelength around 1000 nm). *c* conduit, *m* magnet, *E* EndoVAC; white box indicates the region of interest

Corresponding changes in relative reflectance intensities were seen in the areas depicting the oxygenation status of the hemoglobin (wavelengths between 550 and 600 nm and above 700 nm) (Fig. 4).

### Differences in oxygenation were detectable already during the initial phase of the EndoVAC therapy

**Fig. 5:**
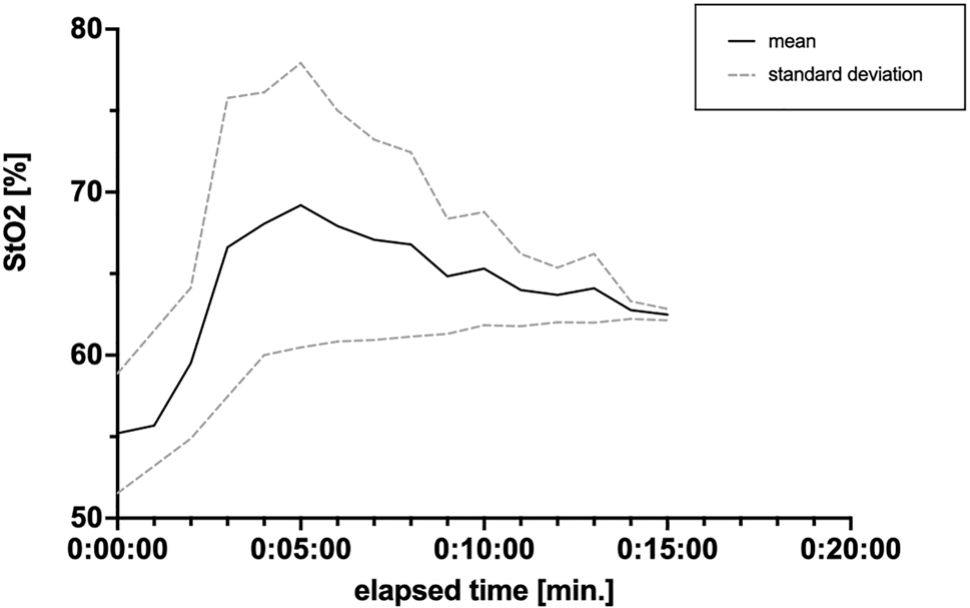
Changes in the tissue oxygenation during the initial phase of EndoVAC therapy with − 200 mmHg pressure. A significant increase in tissue oxygenation from 55.2 ± 3.7% to 69.2 ± 8.7% (*p* = 0.02) is already observed in the first 5 min of the treatment. The solid and dashed lines depict the mean oxygenation values and the standard deviation, respectively

Distinct differences in oxygenation during the initial treatment phase were observed after application of − 200 mmHg pressure. During the first 5 min, the oxygenation significantly increased from 55.2 ± 3.7% to 69.2 ± 8.7% (*p* = 0.02), and then decreased slightly but remained significantly elevated during the entire measurement period (Fig. 5).

### EndoVAC therapy increased the water content of the tissue

A significant increase of tissue water content from 43.3 ± 7.4 to 56.1 ± 10.3 (*p* = 0.02) was observed after 60 min of EndoVAC therapy with − 200 mmHg. After 120 min of therapy with − 200 mmHg, there was a further increase in TWI from 56.1 ± 10.3 3 to 58.7 ± 15.4 (*p* = 0.006). The values after 120 min of EndoVAC therapy with − 200 mmHg were not significantly different from the baseline TWI of 55.5 ± 4.6 (*p* = 0.8). There were no significant differences in TWI under therapy with − 40 and − 125 mmHg (Supplementary Fig. 1. and Supplementary Table 1.). In the control group, there were also no significant changes in the TWI over the entire time of the experiment. The TWI varied over the time from 38.6 ± 12.1 to 44.2 ± 9.8 (*p* = 0.95) (Supplementary Fig. 2 and Supplementary Table 1).

## Discussion

In the present study, we were able to show in a porcine in vivo model, that EndoVAC therapy of hypoperfused gastric conduit increased tissue oxygenation and perfusion at the affected site. Further, we observed that gradually increasing suction pressure had increasing positive effects on tissue oxygenation. Although there are no comparable studies in gastric conduit or esophagus, results from other areas show partially similar trends. The aforementioned trials by Morykwas et al. measured by laser Doppler velocimetry an increased blood flow upon VAC therapy of superficial wounds [26]. Further, Timmers et al. described, that the level of perfusion varied with the amount of negative pressure applied on the healthy skin of a forearm [37]. Too high negative pressure resulted in hypoperfusion of the tissue however. This phenomenon was not observed in our animal trial. On the contrary, the higher the negative pressure was, the more pronounced increase in oxygenation levels was observed in our experiment. However, the highest pressure available in our device was 200 mmHg, which is much lower than the highest negative pressure of 300 mmHg used by Timmers et al. Accordingly, other studies in animal models described that the application of negative pressure in the middle range of the scale, i.e., between 40 and 150 mmHg, had a positive influence on tissue perfusion, especially in the edge area of the wound [26, 38–43]. Our results are further supported by the evidence gathered by Ma et al. in a rat in vivo model of superficial diabetic wound. Ninety-six rats underwent either VAC or gauze treatment of a superficial leg wound. The results revealed that VAC therapy not only increased the blood flow perfusion in the wound area, but also promoted the overexpression of angiogenic factors and maturation of microvessels [44]. This would be interesting to also test in the animal model for gastric conduit with longer lasting experiments in a survival model in the future. However, all comparisons with other studies should be treated with caution, since both the measuring methods and the organs examined differed from these used in our experiment. A comparable study by Scott et al. in a porcine model of anastomotic leakage after Roux-en-Y gastric bypass demonstrated that the histological specimen from EndoVAC treated pigs had lesser degree of severe inflammation and no signs of necrosis or ischemia when compared with the control group [45]. Scott et al. was able to confirm these results also in a porcine model for esophagectomy [46] indirectly supporting our findings of improved tissue oxygenation upon EndoVAC therapy. Further, lower levels of inflammation do not appear to be directly related to bacterial clearance upon VAC therapy. In a prospective controlled trial of 54 patients with a full-thickness wound, no significant difference in total quantitative bacterial load was observed in the interventional group who underwent VAC therapy compared to control group [47]. Also, no changes in bioburden of the wounds upon VAC therapy were observed by Hu et al. despite improved wound healing [48]. Therefore, the action of VAC therapy on bacterial load may be secondary, to other benefits of VAC therapy, which is reflected in success rates in clinical practice. As shown by a recent meta-analysis of five retrospective studies, EndoVAC therapy of anastomotic leak after esophagectomy achieved better outcomes than endoscopically placed self-expandable metal stents (SEMS) alone [49]. These results demonstrated that EndoVAC therapy can be an effective alternative to SEMS for treating anastomotic leaks following esophagectomy. Anastomotic defects were successfully closed in 84–100% of cases treated with EndoVAC, but in only 54–64% after SEMS therapy [11, 15, 19, 50, 51]. After EndoVAC therapy, the likelihood of a successful closure was ninefold higher [49, 52].

Regarding the change in tissue edema, we did not find any comparable study. It can be assumed that our results are the first to provide insight into this field. An increase in Tissue Water Index is consistent with increased levels of tissue perfusion. Further, higher tissue perfusion may be more important than effects of improved venous and lymphatic drainage under EndoVAC therapy but this will have to be investigated in future studies with a longer observation period or a survival experiment. Moreover, in this study, due to technical limitations of the camera system, we were not able to directly examine the esophago-gastric anastomosis. Therefore, the specific influence of EndoVAC therapy on the esophagus remains to be evaluated in the future studies.

In conclusion, our study demonstrated an improved tissue oxygenation of ischemic gastric conduit with EndoVAC therapy with − 125 mg and − 200 mmHg. The changes in tissue edema were decent and detected solely under EndoVAC therapy with − 200 mmHg. This provides insight into pathophysiological mechanisms of EndoVAC therapy in the upper gastrointestinal system and paves the way for further investigations and translation into clinical practice.

### Supplementary Information

Below is the link to the electronic supplementary material.

Supplementary Figure 1 Hyperspectral characterization of tissue water content. (A) The tissue water index (TWI) was measured upon induction of tissue ischemia and after the treatment therapy with with EndoVAC for 120 minutes with pressure of (A) -40 mmHg, (B) -125 mmHg and (C) -200 mmHg. A significant increase of tissue water content from 40.8 ± 7.4 to 59.5 ± 10.3 (p = 0.004) was observed after 60 minutes of EndoVAC therapy with therapy with -200 mmHg. (D+E) Corresponding hyperspectral images of TWI of the gastric conduit. The green and red areas correspond to a low and high TWI respectively.* c, conduit; m, magnet; E, EndoVAC, white box indicates the region of interest.*
Supplementary file1 (JPEG 2586 KB)

Supplementary Figure 2 Tissue edema during induction of tissue ischemia. In the control group tissue ischemia was maintained for the entire duration of the experiment. There were no significant changes in tissue water index (TWI) without EndoVAC therapy after 240 minutes. ns, not significant
Supplementary file2 (JPEG 154 KB)

Supplementary file3 (DOCX 14 KB)

## References

[1] Bray F, Ferlay J, Soerjomataram I, Siegel RL, Torre LA, Jemal A (2018). Global cancer statistics 2018: GLOBOCAN estimates of incidence and mortality worldwide for 36 cancers in 185 countries. CA Cancer J Clin.

[2] Low DE, Kuppusamy MK, Alderson D, Cecconello I, Chang AC, Darling G, Davies A, D’Journo XB, Gisbertz SS, Griffin SM, Hardwick R, Hoelscher A, Hofstetter W, Jobe B, Kitagawa Y, Law S, Mariette C, Maynard N, Morse CR, Nafteux P, Pera M, Pramesh CS, Puig S, Reynolds JV, Schroeder W, Smithers M, Wijnhoven BPL (2019). Benchmarking complications associated with esophagectomy. Ann Surg.

[3] Ye T, Sun Y, Zhang Y, Zhang Y, Chen H (2013). Three-field or two-field resection for thoracic esophageal cancer: a meta-analysis. Ann Thorac Surg.

[4] Goense L, van Dijk WA, Govaert JA, van Rossum PSN, Ruurda JP, van Hillegersberg R (2017). Hospital costs of complications after esophagectomy for cancer. Eur J Surg Oncol EJSO.

[5] Schieman C, Wigle DA, Deschamps C, Nichols Iii FC, Cassivi SD, Shen KR, Allen MS (2012). Patterns of operative mortality following esophagectomy. Dis Esophagus.

[6] Bundred JR, Hollis AC, Evans R, Hodson J, Whiting JL, Griffiths EA (2020). Impact of postoperative complications on survival after oesophagectomy for oesophageal cancer. BJS Open.

[7] Akkerman RDL, Haverkamp L, van Rossum PSN, van Hillegersberg R, Ruurda JP (2015). Long-term quality of life after oesophagectomy with gastric conduit interposition for cancer. Eur J Cancer.

[8] Scarpa M, Saadeh LM, Fasolo A, Alfieri R, Cagol M, Cavallin F, Pinto E, Zaninotto G, Ancona E, Castoro C (2013). Health-related quality of life in patients with oesophageal cancer: analysis at different steps of the treatment pathway. J Gastrointest Surg.

[9] Cavallin F, Pinto E, Saadeh LM, Alfieri R, Cagol M, Castoro C, Scarpa M (2015). Health related quality of life after oesophagectomy: elderly patients refer similar eating and swallowing difficulties than younger patients. BMC Cancer.

[10] Grimminger PP, Goense L, Gockel I, Bergeat D, Bertheuil N, Chandramohan SM, Chen KN, Chon SH, Denis C, Goh KL, Gronnier C, Liu JF, Meunier B, Nafteux P, Pirchi ED, Schiesser M, Thieme R, Wu A, Wu PC, Buttar N, Chang AC (2018). Diagnosis, assessment, and management of surgical complications following esophagectomy. Ann NY Acad Sci.

[11] Brangewitz M, Voigtlander T, Helfritz FA, Lankisch TO, Winkler M, Klempnauer J, Manns MP, Schneider AS, Wedemeyer J (2013). Endoscopic closure of esophageal intrathoracic leaks: stent versus endoscopic vacuum-assisted closure, a retrospective analysis. Endoscopy.

[12] Heits N, Stapel L, Reichert B, Schafmayer C, Schniewind B, Becker T, Hampe J, Egberts J-H (2014). Endoscopic endoluminal vacuum therapy in esophageal perforation. Ann Thorac Surg.

[13] Kuehn F, Schiffmann L, Janisch F, Schwandner F, Alsfasser G, Gock M, Klar E (2016). Surgical endoscopic vacuum therapy for defects of the upper gastrointestinal tract. J Gastrointest Surg.

[14] Laukoetter MG, Mennigen R, Neumann PA, Dhayat S, Horst G, Palmes D, Senninger N, Vowinkel T (2017). Successful closure of defects in the upper gastrointestinal tract by endoscopic vacuum therapy (EVT): a prospective cohort study. Surg Endosc.

[15] Schniewind B, Schafmayer C, Voehrs G, Egberts J, Von Schoenfels W, Rose T, Kurdow R, Arlt A, Ellrichmann M, Jürgensen C, Schreiber S, Becker T, Hampe J (2013). Endoscopic endoluminal vacuum therapy is superior to other regimens in managing anastomotic leakage after esophagectomy: a comparative retrospective study. Surg Endosc.

[16] Schorsch T, Mueller C, Loske G (2014). Endoscopic vacuum therapy of perforations and anastomotic insufficiency of the esophagus. Chirurg.

[17] Tan B, Reddy S, Rashid F, Sujendran V, Safranek P, Hindmarsh A, Hardwick R (2015). Endoscopic transluminal vacuum therapy: an alternative method of treating oesophago-gastric defects. Gut.

[18] Weidenhagen R, Hartl WH, Gruetzner KU, Eichhorn ME, Spelsberg F, Jauch KW (2010). Anastomotic leakage after esophageal resection: new treatment options by endoluminal vacuum therapy. Ann Thorac Surg.

[19] Hwang JJ, Jeong YS, Park YS, Yoon H, Shin CM, Kim N, Lee DH (2016). Comparison of endoscopic vacuum therapy and endoscopic stent implantation with self-expandable metal stent in treating postsurgical gastroesophageal leakage. Medicine.

[20] Ooi G, Burton P, Packiyanathan A, Loh D, Chen R, Shaw K, Brown W, Nottle P (2018). Indications and efficacy of endoscopic vacuum-assisted closure therapy for upper gastrointestinal perforations. Anz J Surg.

[21] Pournaras DJ, Hardwick RH, Safranek PM, Sujendran V, Bennett J, Macaulay GD, Hindmarsh A (2018). Endoluminal vacuum therapy (E-Vac): a treatment option in oesophagogastric surgery. World J Surg.

[22] Bludau M, Fuchs HF, Herbold T, Maus MKH, Alakus H, Popp F, Leers JM, Bruns CJ, Holscher AH, Schroder W, Chon SH (2018). Results of endoscopic vacuum-assisted closure device for treatment of upper GI leaks. Surg Endosc.

[23] Mencio MA, Ontiveros E, Burdick JS, Leeds SG (2018). Use of a novel technique to manage gastrointestinal leaks with endoluminal negative pressure: a single institution experience. Surg Endosc Interv Tech.

[24] Manfredi MA, Clark SJ, Staffa SJ, Ngo PD, Smithers CJ, Hamilton TE, Jennings RW (2018). Endoscopic esophageal vacuum therapy: a novel therapy for esophageal perforations in pediatric patients. J Pediatr Gastroenterol Nutr.

[25] Gubler C, Vetter D, Schmidt HM, Muller PC, Morell B, Raptis D, Gutschow CA (2018). Preemptive endoluminal vacuum therapy to reduce anastomotic leakage after esophagectomy: a game-changing approach?. Esophagus.

[26] Morykwas MJ, Argenta LC, Shelton-Brown EI, McGuirt W (1997). Vacuum-assisted closure: a new method for wound control and treatment: animal studies and basic foundation. Ann Plast Surg.

[27] Morykwas MJ, Faler BJ, Pearce DJ, Argenta LC (2001). Effects of varying levels of subatmospheric pressure on the rate of granulation tissue formation in experimental wounds in swine. Ann Plast Surg.

[28] Kamolz LP, Andel H, Haslik W, Winter W, Meissl G, Frey M (2004). Use of subatmospheric pressure therapy to prevent burn wound progression in human: first experiences. Burns.

[29] Kamarajah SK, Lin A, Tharmaraja T, Bharwada Y, Bundred JR, Nepogodiev D, Evans RPT, Singh P, Griffiths EA (2020). Risk factors and outcomes associated with anastomotic leaks following esophagectomy: a systematic review and meta-analysis. Dis Esophagus Off J Int Soc Dis Esophagus.

[30] Li S-J, Wang Z-Q, Li Y-J, Fan J, Zhang W-B, Che G-W, Liu L-X, Chen L-Q (2017). Diabetes mellitus and risk of anastomotic leakage after esophagectomy: a systematic review and meta-analysis. Dis Esophagus Off J Int Soc Dis Esophagus.

[31] Mengardo V, Pucetti F, Mc Cormack O, Chaudry A, Allum WH (2018). The impact of obesity on esophagectomy: a meta-analysis. Dis Esophagus.

[32] Kassis ES, Kosinski AS, Ross P, Koppes KE, Donahue JM, Daniel VC (2013). Predictors of anastomotic leak after esophagectomy: an analysis of the society of thoracic surgeons general thoracic database. Ann Thorac Surg.

[33] Zheng Q-F, Wang J-J, Ying M-G, Liu S (2013). Omentoplasty in preventing anastomotic leakage of oesophagogastrostomy following radical oesophagectomy with three-field lymphadenectomy. Eur J Cardiothorac Surg.

[34] Nickel F, Studier-Fischer A, Özdemir B, Odenthal J, Müller LR, Knoedler S, Kowalewski KF, Camplisson I, Allers MM, Dietrich M, Schmidt K, Salg GA, Kenngott HG, Billeter AT, Gockel I, Sagiv C, Hadar OE, Gildenblat J, Ayala L, Seidlitz S, Maier-Hein L, Müller-Stich BP (2023). Optimization of anastomotic technique and gastric conduit perfusion with hyperspectral imaging and machine learning in an experimental model for minimally invasive esophagectomy. Eur J Surg Oncol J Eur Soc Surg Oncol Br Assoc Surg Oncol.

[35] Studier-Fischer A, Seidlitz S, Sellner J, Bressan M, Özdemir B, Ayala L, Odenthal J, Knoedler S, Kowalewski K-F, Haney CM, Salg G, Dietrich M, Kenngott H, Gockel I, Hackert T, Müller-Stich BP, Maier-Hein L, Nickel F (2023). HeiPorSPECTRAL—the Heidelberg Porcine HyperSPECTRAL imaging dataset of 20 physiological organs. Sci Data.

[36] Studier-Fischer A, Seidlitz S, Sellner J, Özdemir B, Wiesenfarth M, Ayala L, Odenthal J, Knödler S, Kowalewski KF, Haney CM, Camplisson I, Dietrich M, Schmidt K, Salg GA, Kenngott HG, Adler TJ, Schreck N, Kopp-Schneider A, Maier-Hein K, Maier-Hein L, Müller-Stich BP, Nickel F (2022). Spectral organ fingerprints for machine learning-based intraoperative tissue classification with hyperspectral imaging in a porcine model. Sci Rep.

[37] Timmers MS, Le Cessie S, Banwell P, Jukema GN (2005). The effects of varying degrees of pressure delivered by negative-pressure wound therapy on skin perfusion. Ann Plast Surg.

[38] Wackenfors A, Gustafsson R, Sjögren J, Algotsson L, Ingemansson R, Malmsjö M (2005). Blood flow responses in the peristernal thoracic wall during vacuum-assisted closure therapy. Ann Thorac Surg.

[39] Wackenfors A, Sjögren J, Gustafsson R, Algotsson L, Ingemansson R, Malmsjö M (2004). Effects of vacuum-assisted closure therapy on inguinal wound edge microvascular blood flow. Wound Repair Regen.

[40] Petzina R, Gustafsson L, Mokhtari A, Ingemansson R, Malmsjö M (2006). Effect of vacuum-assisted closure on blood flow in the peristernal thoracic wall after internal mammary artery harvesting. Eur J Cardiothorac Surg.

[41] Borgquist O, Ingemansson R, Malmsjö M (2010). Wound edge microvascular blood flow during negative-pressure wound therapy: examining the effects of pressures from –10 to –175 mmHg. Plast Reconstr Surg.

[42] Ichioka S, Watanabe H, Sekiya N, Shibata M, Nakatsuka T (2008). A technique to visualize wound bed microcirculation and the acute effect of negative pressure. Wound Repair Regen.

[43] Chen S-Z, Li J, Li X-Y, Xu L-S (2005). Effects of vacuum-assisted closure on wound microcirculation: an experimental study. Asian J Surg.

[44] Ma Z, Li Z, Shou K, Jian C, Li P, Niu Y, Qi B, Yu A (2017). Negative pressure wound therapy: Regulating blood flow perfusion and microvessel maturation through microvascular pericytes. Int J Mol Med.

[45] Scott RB, Ritter LA, Shada AL, Feldman SH, Kleiner DE (2016). Endoluminal vacuum therapy for gastrojejunal anastomotic leaks after Roux-en-Y gastric bypass: a pilot study in a swine model. Surg Endosc.

[46] Scott R, Ritter L, Shada A, Feldman S, Kleiner D (2017). Endoluminal vacuum therapy for ivor lewis anastomotic leaks: a pilot study in a swine model. Clin Transl Sci.

[47] Moues CM, Vos MC, Van den Bemd G, Stijnen T, Hovius SER (2004). Bacterial load in relation to vacuum-assisted closure wound therapy: a prospective randomized trial. Wound Repair Regen.

[48] Hu K, Zhang H, Zhou F, Yao G, Shi J, Wang L, Hou Z (2009). A comparative study of the clinical effects between two kinds of negative-pressure wound therapy. Zhonghua Shao Shang Za Zhi Zhonghua Shaoshang Zazhi Chin J Burns.

[49] Scognamiglio P, Reeh M, Karstens K, Bellon E, Kantowski M, Schön G, Zapf A, Chon S-H, Izbicki JR, Tachezy M (2020). Endoscopic vacuum therapy versus stenting for postoperative esophago-enteric anastomotic leakage: systematic review and meta-analysis. Endoscopy.

[50] Mennigen R, Harting C, Lindner K, Vowinkel T, Rijcken E, Palmes D, Senninger N, Laukoetter MG (2015). Comparison of endoscopic vacuum therapy versus stent for anastomotic leak after esophagectomy. J Gastrointest Surg.

[51] Berlth F, Bludau M, Plum PS, Herbold T, Christ H, Alakus H, Kleinert R, Bruns CJ, Hölscher AH, Chon S-H (2019). Self-expanding metal stents versus endoscopic vacuum therapy in anastomotic leak treatment after oncologic gastroesophageal surgery. J Gastrointest Surg.

[52] Rausa E, Asti E, Aiolfi A, Bianco F, Bonitta G, Bonavina L (2018). Comparison of endoscopic vacuum therapy versus endoscopic stenting for esophageal leaks: systematic review and meta-analysis. Dis Esophagus.

[53] Percie du Sert N, Ahluwalia A, Alam S, Avey MT, Baker M, Browne WJ, Clark A, Cuthill IC, Dirnagl U, Emerson M, Garner P, Holgate ST, Howells DW, Hurst V, Karp NA, Lazic SE, Lidster K, MacCallum CJ, Macleod M, Pearl EJ, Petersen OH, Rawle F, Reynolds P, Rooney K, Sena ES, Silberberg SD, Steckler T, Würbel H (2020). Reporting animal research: Explanation and elaboration for the ARRIVE guidelines 2.0. PLoS Biol.

