# Supplementary figures and images for: Effects of endoluminal vacuum sponge therapy on the perfusion of gastric conduit in a porcine model for esophagectomy
Source: Surg Endosc. 2024 Jan 5;38(3):1422–31. doi: 10.1007/s00464-023-10647-0 (PMC10881612; doi:10.1007/s00464-023-10647-0)

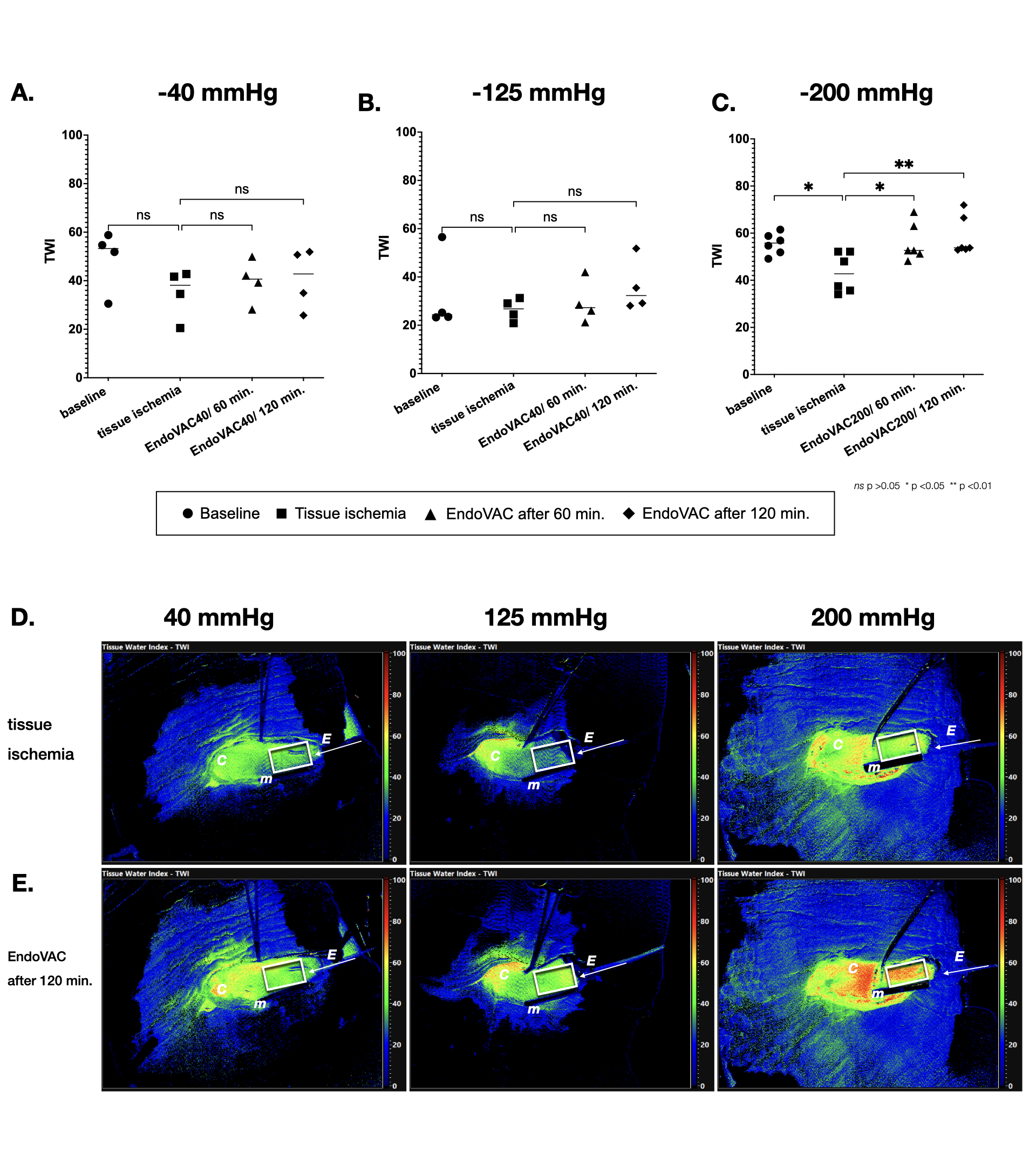

Supplement: Supplementary file 1 — Supplementary Figure 1 Hyperspectral characterization of tissue water content. (A) The tissue water index (TWI) was measured upon induction of tissue ischemia and after the treatment therapy with with EndoVAC for 120 minutes with pressure of (A) -40 mmHg, (B) -125 mmHg and (C) -200 mmHg. A significant increase of tissue water content from 40.8 ± 7.4 to 59.5 ± 10.3 (p = 0.004) was observed after 60 minutes of EndoVAC therapy with therapy with -200 mmHg. (D+E) Corresponding hyperspectral images of TWI of the gastric conduit. The green and red areas correspond to a low and high TWI respectively. c, conduit; m, magnet; E, EndoVAC, white box indicates the region of interest. Supplementary file1 (JPEG 2586 KB) [file 464_2023_10647_MOESM1_ESM.jpeg]

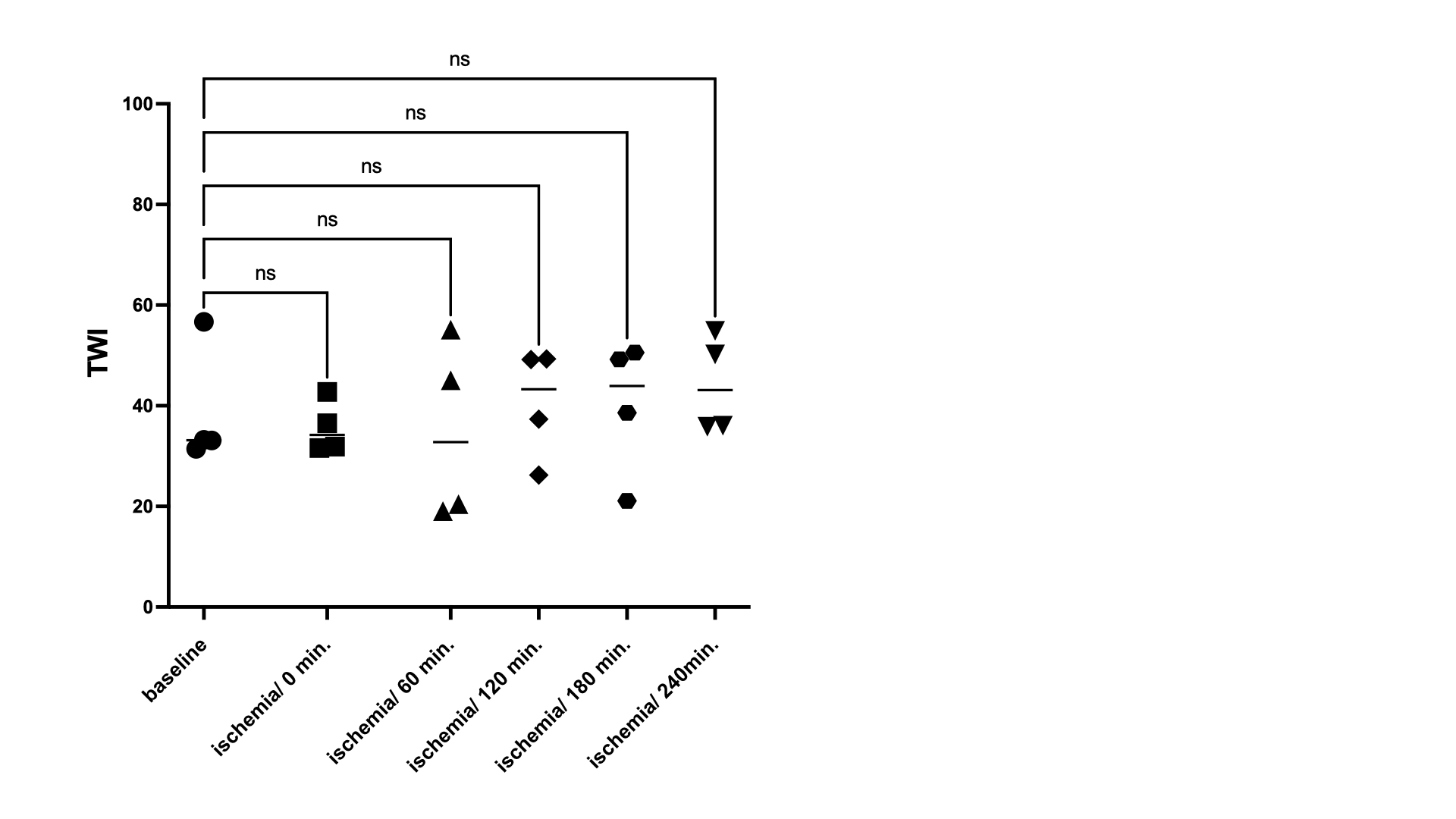

Supplement: Supplementary file 2 — Supplementary Figure 2 Tissue edema during induction of tissue ischemia. In the control group tissue ischemia was maintained for the entire duration of the experiment. There were no significant changes in tissue water index (TWI) without EndoVAC therapy after 240 minutes. ns, not significant Supplementary file2 (JPEG 154 KB) [file 464_2023_10647_MOESM2_ESM.jpeg]
