# Supplementary material for: Effects of endoluminal vacuum sponge therapy on the perfusion of gastric conduit in a porcine model for esophagectomy
Source: Surg Endosc. 2024 Jan 5;38(3):1422–31. doi: 10.1007/s00464-023-10647-0 (PMC10881612; doi:10.1007/s00464-023-10647-0)
Supplement: Supplementary file 3 — Supplementary file3 (DOCX 14 KB) [file 464_2023_10647_MOESM3_ESM.docx]

|  | **Group A**  **(-40 mmHg)** | **Group B**  **(-125 mmHg)** | **Group C**  **(-200 mmHg)** |  | **Group D**  **(no EndoVAC)** |
| --- | --- | --- | --- | --- | --- |
| n | 4 | 4 | 6 | n | 4 |
| Baseline | 49.0 ± 12.6 | 32.1 ± 16.3 | 55.5 ± 4.6 | Baseline | 38.6 ± 12.1 |
| Tissue ischemia after |  |  |  | Tissue ischemia after |  |
| 60 min. | 36.3 ± 8.7 | 29.1 ± 10.3 | 45.7 ± 12.9 | 60 min. | 34.9 ± 18.0 |
| 120 min. | 34.9 ± 10.3 | 28.9 ± 5.8 | 40.8 ± 7.4 | 120 min. | 40.5 ± 11.1 |
| EndoVAC after 60 min. | 39.8 ± 9.0 | 29.5 ± 8.9 | 59.5 ± 10.3 | 180 min. | 39.9 ± 13.6 |
| EndoVAC after 120 min. | 40.8 ± 12.7 | 36.1 ± 11.0 | 58.6 ± 8.4 | 240 min. | 44.2 ± 9.8 |

**Supplementary Table 1.** **Tissue Water Index (TWI).** The index values are depicted as means and their standard deviations.
